# Reply to Klein et al.: The importance of aerosol pH for airborne respiratory virus transmission

**DOI:** 10.1073/pnas.2212556119

**Published:** 2022-08-29

**Authors:** Henry P. Oswin, Allen E. Haddrell, Mara Otero-Fernandez, Jamie F. S. Mann, Tristan A. Cogan, Thomas G. Hilditch, Jianghan Tian, Dan Hardy, Darryl J. Hill, Adam Finn, Andrew D. Davidson, Jonathan P. Reid

**Affiliations:** ^a^School of Chemistry, University of Bristol, Bristol BS8 1TS, United Kingdom;; ^b^Bristol Veterinary School, University of Bristol, Bristol BS40 5DU, United Kingdom;; ^c^School of Cellular and Molecular Medicine, University of Bristol, Bristol BS8 1TD, United Kingdom

We are grateful to Klein et al. for their letter (1) reinforcing the importance of understanding the pH of exhaled aerosol when assessing the airborne survival of respiratory pathogens. The pH of aerosol particles can evolve rapidly during transport, reflecting changes in particle composition coupled to changes in gas-phase composition. Studies have identified that exhaled breath condensates are alkaline (up to pH 8.5) from healthy individuals (2), and we have concluded that exhaled droplets can become alkaline (up to pH 11) with consequences for infectivity of severe acute respiratory syndrome coronavirus 2 (3). Klein et al. suggests that condensable acidic species (e.g., nitric acid) can lower the pH to as low as three, leading to conditions that compromise viral infectivity. The microphysical model of Klein et al. could provide important mechanistic insights into measurements of airborne survival, although we respond to some of their assertions.

## Generalization of Tendency to Alkalinity.

Although we suggested that acidic vapors can lower the pH of respiratory aerosol (3), the evaporation of dissolved carbon dioxide and loss of bicarbonate can increase the pH at early times, responding to the change in gas-phase CO_2_ concentration following expiration. Figure 1 of Klein et al. (1) confirms that there is a substantial increase in pH over a timescale from <1 s to ∼10 s (not 20 min as Klein et al. suggests) for both particle sizes and trace gas compositions modeled. Whether the aerosol is alkaline or acidic is a matter of time, and both occur. The pH rise may be transient and localized, lasting from as little as 10 s to a day or longer, but the impact on viral infectivity must be considered.

## Sensitivity to Gas and Particle Phase Compositions.

Klein et al. (1) demonstrates that the persistence of alkaline conditions is dependent on both the gas- and the particle-phase compositions (e.g., concentration of bicarbonate) and, thus, any initialization conditions set in any model. Although the artificial saliva formulation of Woo et al. (4) contains only ∼10% of the bicarbonate in the fluid we use (minimal essential media [MEM]), real saliva can be extremely variable in bicarbonate concentration, with values as high as 3.66 g/L reported (5), 2 times higher than MEM. We recommend a comprehensive model sensitivity analysis of temporal trends in pH to compositional inputs before final equilibrated values are assumed to determine ultimate viral survival.

## Relative Importance of 2- and 25-μm-Radius Particles.

The contributions of particles of different sizes to transmission remains unresolved, with only a few studies attempting to fractionate infectious virus by particle size (6, 7). Prather et al. (8) conclude that all particles of <100-μm diameter show similar aerodynamic behavior and can be advected around indoor spaces. Droplets initially 25 μm in radius evaporate to <6 μm in <5 s, as seen by Klein et al. (1) and our own work (9). Thus, we cannot agree with Klein et al.’s implied irrelevance of this larger particle size. We can agree that, for larger particles, “gas-to-particle mass transfer kinetics is slow enough so that about a day is required … to condense and make the particle acidic.” The early tendency to alkaline pH will be extremely important.
